# Influence of Different Exercise Types on Health-Related Quality-of-Life in Men With Depressive Disorder in South Korea

**DOI:** 10.3389/fpubh.2022.811168

**Published:** 2022-03-14

**Authors:** Kyungjin Kim, Kyo-Man Koo

**Affiliations:** ^1^Department of Adapted Physical Activity, Korea Nazarene University, Cheonan-si, South Korea; ^2^Department of Adapted Physical Education, Baekseok University, Cheonan-si, South Korea

**Keywords:** exercise, health-related quality of life, men, depressive disorder, South Korea

## Abstract

The aim of the study was to determine the influence of different exercise types on health-related quality of life (QOL) in men with depressive disorder (DD) in South (S) Korea. The data of 385 men aged 19 with DD were collected in S. Korea. The Euro Quality of Life 5 Dimensions (EQ-5D) index and Korea National Health and Nutrition Examination Survey (KNHANES) questionnaires were used to establish the purpose of this study. Furthermore, the complex sampling model was applied to investigate the influence of different exercise types on health-related QOL in participants. When reviewing the outcomes, the strength exercise and walking had significant influences on health-related QOL in men with DD in S. Korea. However, the flexibility exercise did not have a significant influence on them. Based on the results, strength exercise and walking were effective exercise types to increase levels of health-related QOL in men with DD in S. Korea.

## Introduction

In modern society, not only are physical health problems recognized as important, but mental health issues are recognized as important as well. According to the WHO (1), mental health is one of the integral elements of health and wellbeing (1). Furthermore, it refers to a state in which one can do productive work and contribute to the community while overcoming the stress that occurs in daily life. However, many individuals receive a lot of stress from various factors, which can lead to depressive disorder (DD). The DD is a type of mood disorder, and the main symptoms of depression are sadness, loneliness, and low motivation (2). It is the most common disorder worldwide and high frequency of comorbidity (3, 4). For example, it is one of the ten global burdens of disease for humans and has affected more than 260 million people in 2020 (5). It was also projected to become the second public health problem after 2020 (6). Hence, the DD is a serious challenge worldwide.

In South (S) Korea, the DD is the strongest risk factor for suicide and is more likely to be caused by severe and pervasive stress (7–9). The suicide rate per 100,000 persons was 30, which is the highest among Organization for Economic Cooperation and Development (OECD) countries (10). In the case of depression diagnosis, women are reported to have higher rates of depression (e.g., about a quarter of Korean women suffer from depressive symptoms) than men (11), but men tend to hide their mental disease particularly in S. Korea (12). Unlike women, it is underestimated that DD in men is not expressed in an emotional way. Nevertheless, the suicide rate related to a depressive symptom was 1.8 times higher in men than in women in S. Korea (12). Since suicide rates due to DD are higher in men than in women, DD in men should not be underestimated (13).

Male college students in S. Korea are experiencing various stressors, such as work, army service, and study; as a result, a significant number of them have depressive episodes (14–16). Middle-aged men are under a lot of stress physically and mentally because of cognitive decline, family problems, economic challenges, heavy responsibilities in social life, and anxiety due to competition with the younger generation (17). Moreover, it was reported that middle-aged men showed that low quality of life (QOL) and that QOL decreased when the level of depressive disorder was high (17). According to the national report data of S. Korea, about 28% of the elderly people (e.g., 65 years old and above) fall into the category of diagnosis of depression, and the number of elderly people with DD is increasing every year (e.g., increasing more than 60% in the past 5 years) (18). In recent years, the number of male elderly people who have depressive symptoms in S. Korea is increasing, and male elderly people with DD are linked to lower rates of QOL (19).

Individuals with DD are known to be less physically active (20). According to Jerstad et al. (21), adolescents who are less physically active were more likely to suffer from depressive events. Especially due to the lack of physical activity (PA) from men with DD, there is an increase in pessimistic and self-critical thinking (22). The elderly patients experiencing DD were expected to exhibit more undesirable health behaviors, such as less PA and more smoking (23). In addition, they feel negative about their present and future situations and lead isolated life (21). These factors were found to have a considerable effect on the health-related QOL. WHO reported that the health-related QOL included not only physical, but also emotional, social, and mental functions, and it can be defined as health, death, and expectation of life on QOL. All of these can be improved through exercise as reported literature suggested (5).

According to earlier studies (24, 25), participation in exercise was known to reduce depressive indications not only in depressed patients but also in healthy individuals. It was also reported that exercise is effective in preventing complications related to mental health. For instance, people's psychological changes might be shifted in a positive direction through exercise. Furthermore, continuous exercise relieved depression and stress in middle age and increases health and QOL (26, 27). Thus, the health-related QOL of people with DD might be closely associated with exercise. However, most studies have mainly focused on the health-related QOL in women with DD, and studies related to DD in men are relatively unnoticed and rarely conducted. Some studies investigated the effects of PA of pregnant women with DD on QOL (28–30). Several studies noted that increased exercise can help to prevent young or elderly women from experiencing depressive symptoms (31–33). In studies related to men with DD, Vancampfort et al. (34) reported that men with major DD were less physically active and had low levels of physical fitness and QOL. Chae et al. (35) investigated that there was an effect of exercise on the DD, and moderate-intensity walking was effective for depressive indicators.

Although exercise is a significant factor to prevent depressive episodes in men and improve the health-related QOL, there is limited evidence to provide information that exercise is valuable for them (5, 20–23). In addition, most of the researchers determined the effect on health-related QOL through limited exercise type (24–35). Consequently, the purpose of this study was to investigate the influence of different exercise types (e.g., flexibility exercise, strength exercise, and walking) on the health-related QOL in men with DD in S. Korea.

## Methods

### Participants

The aim of the study was to investigate the influence of different exercise types (e.g., flexibility exercise, strength exercise, and walking) on the health-related QOL in men with DD in S. Korea, applying a secondary analysis by using the raw data of the Korea National Health and Nutrition Examination Survey (KNHANES). There was the recommendation of the WHO to conduct a survey, being the authorization of the KNHANES from the institutional review board (IRB) in Korea Centers for Disease Control and Prevention Agency. The raw data (e.g., the 4th, 5th, 6th; 2007–2015) of the KNHANES were vertically combined to improve statistical power and included many participants (e.g., 24,871 people from the 4th data in 2007–2009; 31,596 people from the 5th data in 2010–2012; 22,948 people from the 6th data in 2013–2015). Additionally, the data are representative statistical data of S. Korea, conducted every year and sampled by the laminar collection system extraction process. In this study, there was a total of 385 (unweighted cases) of men in S. Korea, who were over 19 years old, and who answered with current depression. The demographic characteristics of participants (i.e., men with DD in S. Korea) are in Supplementary Table 1.

### Research Instrument

The International Physical Activity Questionnaire (IPAQ) includes 4 sets of questionnaires. The purpose of IPAQ is to present a conventional tool that can be used to have international data on health-related PA. The IPAQ was a standard to measure the level of physical activity in participants aged 16–65 and helped to begin the KNHANES. There are two types of questionnaires, such as long-form and short-form questionnaires, which are considered as culturally different (31). The KNHANES was based on the short form of the IPAQ and was calculated the validation (i.e., ranging from 1.12 to 0.57) by Pearson correlation coefficient (32). The correlation (i.e., Spearman coefficient 0.27) between the KNHANES and IPAQ was calculated by the accelerometer (33). The KNHANES collects data on demographic characteristics (e.g., age, ownership of a house, degree of stress recognition, economic activity, and activity restriction), health level (e.g., health behavior survey, health interview survey, and household survey), medical examinations, and nutrition information of participants aged 19 years or older. In this study, two areas (i.e., demographic characteristics, health level) of KNHANES were used to determine the aim of the study. Especially, the participants were investigated for activity restrictions, economic activities, education, medical use, morbidity, and physical activities in the health interview and health level survey. Furthermore, there were alcohol, mental health, oral health, safety awareness, and smoking in the health behavior survey (24, 25). This presentive questionnaire has helped to setup the national health policy in S. Korea since 1988.

### Research Variables

The variables of the study were preferred as different exercise types (e.g., flexibility exercise, strength exercise, and walking) on health-related QOL. The research variables were categorized by the process of analysis and review of prior studies (34, 35). Further, the different exercise types were selected by using the National Health and Nutrition Examination Guidelines (35). Consequently, the KNHANES Guidelines helped to categorize variables of this study based on the purpose of the study. Response categories of questions (e.g., flexibility and strength exercise) of variables were from original responses (e.g., 1 = Not once, 2 = 1 day, 3 = 2 days, 4 = 3 days, 5 = 4 days, 6 = 5 days, and more) to modified responses (e.g., 1 = 5 days and more, 2 = 3–4 days, 3= 1–2 days, and 4 = Not once). Response category of walking was from original responses (e.g., 1 = Not once, 2 = 1 day, 3 = 2 days, 4 = 3 days, 5 = 4 days, 6 = 5 days, 7 = 6 days, and 8 = 7 days) to modified responses (e.g., 1 = 5 days and more, 2 = 3–4 days, 3= 1–2 days, and 4 = Not once). Since the scale of response categories in each variable was different, the response categories were modified for common statistical work and analysis. The questions of research variables of each exercise type in KNHANES are in Supplementary Table 2.

The dependent variable was the health-related QOL of men with DD in S. Korea and was analyzed by using the Euro Quality of Life 5 Dimensions (EQ-5D index score). EQ-5D consists of five areas, such as self-care, pain/discomfort, anxiety/depression, mobility, and usual activity. There is a 3-point scale in responses to each question in EQ-5D. The questions of dependent variables of each area in EQ-5D are in Supplementary Table 2. According to the formula of QOL in EQ-5D, the different answers and levels of health can be measured as a total of 3^5^ = 243 (see Supplementary Table 3).

### Data Analysis

The design of the complex sampling recommended in the KNHANES Guidelines (35, 36) was used to investigate the influence of different exercise types (e.g., flexibility exercise, strength exercise, and walking) on health-related QOL in men with DD in S. Korea. The data of the questionnaire from 2007 to 2015 were vertically combined and estimating the dispersion layer used to designate the stratification variable. The integration weights for analysis were 0.5/8.5 in 2007 and 1/8.5 from 2008 to 2015. The KNHANES and EQ-5D questionnaires were modified from original responses for data analysis. The complex sampling in frequency analysis was used to determine the demographic characteristics. Furthermore, the research variables were analyzed by using the complex sample general linear model and the significance level was *p* < 0.05. The complex sample general linear model was a procedure to conduct the linear regression analysis, variance analysis, and covariance analysis for samples drawn by methods of complex sampling. All missing data in elements were included to avoid the biased events in the dispersion estimator and estimation after the elimination of missing data, and the value of missing users was adjusted to acceptable values. All statistical data in this study were analyzed by SPSS 21.0 version.

## Results

### Influence of Different Exercise Types (e. g., Flexibility Exercise, Strength Exercise, and Walking) on the Health-Related QOL in Men With Depressive Disorder in S. Korea

The influence of different exercise types (e.g., flexibility exercise, strength exercise, and walking) on the levels of the health-related QOL in men with DD in S. Korea was analyzed by using the complex sample general linear model. It was estimated that 40.1% could explain the level of health-related QOL in the case of men with DD in S. Korea. Based on the outcomes of the rate of participation by different exercise types (e.g., flexibility exercise, strength exercise, and walking), it was a significant statistical difference in the group who had in the 5 days from the group who did not have strength exercise at all. In walking, it was a significant statistical difference in the group who had in 1–2 days from the group who did not have in walking at all. On the other hand, there was not a significantly statistical difference in flexibility exercise. The outcomes of the influence of different exercise types on health-related QOL in men with DD in S. Korea are shown in Supplementary Table 4.

## Discussion

The aim of this study was to identify that there are differences between the influence of different exercise types (e.g., flexibility exercise, strength exercise, and walking) on the health-related QOL in men (19 years old or older) with DD in S. Korea. The design of the complex sampling was conducted to determine the influence of different exercise types on health-related QOL in men with DD. Based on the outcomes of this study, men with DD in S. Korea who participated in strength exercise for 5 days or more a week had higher scores of health-related QOL than the others who had not participated in strength exercise at all. In addition, men with DD in S. Korea who participated in walking for 1–2 days had higher scores of health-related QOL than the others who had not participated in walking at all. However, participants in flexibility exercise did not have statistically significant differences in health-related QOL.

In this study, there was not a significant influence of flexibility exercise on health-related QOL of men with DD in S. Korea. Many men tend not to like flexibility exercises because men think that flexibility exercises are boring because they think those are physical exercises that only women perform. This finding was related to results by Kim and Koo (37), who presented that flexibility exercise was thought to limit one's body functions and eventually lead to depression symptoms. In addition, a study (38) reported that high-frequency exercise, or exercise that is not always enjoyable, provided psychological burden and stress, which can cause negative consequences for QOL. However, flexibility exercise helps to improve the quality of rest and sleep, which can provide psychological stability and release stress (39). Many women in particular show improved balance, increased self-esteem, and reduced pain with flexibility exercises, such as yoga, Pilates, and stretching. Flexibility exercise enhances the health-related QOL with positive effects on the physical and mental components (40). Hence, flexibility exercise might be necessary to perform with psychological intervention to minimize depression and antipathy when men with DD participate in flexibility exercises even though this study did not have a significant influence of flexibility exercise on them.

In strength exercise, there was a significant influence in in group who participated in strength exercise for 5 days or over had higher levels of health-related QOL than the other groups. Many researchers provided evidence about the positive influences of strength exercise on health-related QOL in men (41–43). It increases energy expenditure and increases muscle mass, which helps to maintain a healthy life. In addition, Sayer et al. (44) reported that there was an effect of the grip strength on health-related QOL in the elderly. These researchers stated that individuals can prevent muscle loss when participating in strength exercise programs. Muscle mass decreases with age, and this situation is especially prevalent in men. Men suffering from depressive symptoms were known to be less physically active, and those who are less physically active were reported to have lower bone density. When bone density is low, the possibility of fracture increases, which is a factor that lowers health-related QOL in men with DD. Since strength exercise increases bone density, it can ultimately improve the health-related QOL (22).

On the other hand, Koo and Kim (45) suggested that strength exercise did not provide the positive effects on health-related QOL of Korean women with the DD because it might create a negative active state when the emotional state of women is negative. Furthermore, it was reported that women had difficulty in strength exercise when they were stressed by the frequency of strength exercise, which resulted in negative results. Although it was difficult for women with DD to do strength exercise, it was an effective exercise for men with the depressive disorder to improve their confidence through strength exercise and for the elderly to enhance their level of PA (46, 47). Thus, strength exercise with high frequency (i.e., 5 days or more) should be considered for men with DD in S. Korea because it can improve levels of PA and mental health, which will support health-related QOL.

In the present study, there was a significant influence in the group who participated in walking for 1–2 days had higher levels of health-related QOL than the others. This result is in line with previous studies (48, 49), which stated that after surgery or illness, as people often experience emotional illness and depressive episodes. As a result of an experiment on these people, it was reported that health-related QOL was improved tremendously after walking. This is because walking is not only an exercise with daily activity but also has the effect of refreshing the mood (49, 50). Those who practice active exercise, such as walking, have low psychological anxiety and positively respond to stressful situations to increase their scores of QOL and evaluate their health status positively. This will eventually lead to an opportunity to improve health-related QOL. Furthermore, aerobic exercise, such as walking, can increase cardiorespiratory endurance and can efficiently control fat, which helps maintain health (51). However, in this study, only low-frequency walking (i.e., 1–2 days) was shown to have an effect on health-related QOL, so the frequency of exercise should be considered.

Therefore, these findings were associated with outcomes by an earlier study (37, 45), that are dependent on the frequency of exercise. The effect of exercise may appear positively, or rather, the high frequency may have a negative effect by causing stress. In particular, the type and frequency of exercise should be carefully provided to men with DD.

## Research Limitations

This research has some limitations. First, the results of this study cannot be generalized because the subjects of this study were men in S. Korea aged 19 and the only results of Korean studies are included. Studies including the results of other countries should be conducted to generalize this study. Second, this study could not be used for all Koreans because the subjects were limited to Korean men over the age of 19. Therefore, in future research, it is necessary to conduct research that includes Korean women's data. Third, the raw data (e.g., the 4th, 5th, 6th; 2007–2015) of the KNHANES were only used in this study since a new version of questions in the KNHANES was used after 2015. Consequently, a future study should be examined to determine the effect of exercise on health-related QOL by using the new version of questions in the KNHANES. Lastly, since the data in this study were extracted only through specific exercise (e.g., flexibility exercise, strength exercise, and walking) and exercise frequency, future studies need more data on various exercise types and exercise intensity.

## Conclusions

The purpose of this study was to investigate the influence of different exercise types (e.g., flexibility exercise, strength exercise, and walking) on the health-related QOL in men with DD in S. Korea. The main conclusions drawn were that strength exercise and walking had a significant influence on health-related QOL in men with DD in S. Korea. However, flexibility exercise did not have a significant influence on health-related QOL in men with DD in S. Korea. Potential research should be considered to investigate the influence of various exercise types and exercise intensity on health-related QOL in men with DD.

## Data Availability Statement

The original contributions presented in the study are included in the article/Supplementary Material, further inquiries can be directed to the corresponding author.

## Ethics Statement

The study was conducted according to the guidelines of the Declaration of Helsinki. Ethical review and approval were waived for this study because KNHANES V-VII was conducted by obtaining the approval of Research Ethics Review Committee of Korea Disease Control and Prevention Agency (2010-02CON-21-C; 2011-02CON-06-C; 2012-01EXP-01-2C; 2013-07CON-03-4C; 2013-12EXP-03-5C; and 2018-01-03-P-A). Written informed consent for participation was not required for this study in accordance with the national legislation and the institutional requirements.

## Author Contributions

K-MK and KK: conceptualization, methodology, software, investigation, resources, and data curation. K-MK: formal analysis. KK: writing-review and editing. Both authors have read and agreed to the published version of the manuscript.

## Conflict of Interest

The authors declare that the research was conducted in the absence of any commercial or financial relationships that could be construed as a potential conflict of interest.

## Publisher's Note

All claims expressed in this article are solely those of the authors and do not necessarily represent those of their affiliated organizations, or those of the publisher, the editors and the reviewers. Any product that may be evaluated in this article, or claim that may be made by its manufacturer, is not guaranteed or endorsed by the publisher.
