# Supplementary material for: Influence of Different Exercise Types on Health-Related Quality-of-Life in Men With Depressive Disorder in South Korea
Source: Front Public Health. 2022 Mar 14;10:811168. doi: 10.3389/fpubh.2022.811168 (PMC8964042; doi:10.3389/fpubh.2022.811168)
Supplement: Supplementary file 1 [file Table_1.docx]

**Table 1.** Demographic characteristics of men with DD in S. Korea

| **Characteristics.** | | **Unweighted *N*** | | | **Weighted *N*** | | **% (Weighted)** | |
| --- | --- | --- | --- | --- | --- | --- | --- | --- |
| Age | 19–29 | | 31 | 41,684.832 | | 15.9 | |  |
|  | 30–39 | | 24 | 26,806.406 | | 10.2 | |  |
|  | 40–49 | | 54 | 55,324.955 | | 21.1 | |  |
|  | 50–59 | | 80 | 70,717.463 | | 26.9 | |  |
|  | 60–69 | | 84 | 35,832.919 | | 13.7 | |  |
|  | 70≤ | | 85 | 32,082.256 | | 12.2 | |  |
| Ownership of  House | 0 | | 137 | 120,807.277 | | 46.4 | |  |
|  | 1 | | 179 | 113,126.287 | | 43.5 | |  |
|  | 2 more | | 41 | 26,279.751 | | 10.1 | |  |
| Degree of Stress Recognition | Extremely | | 69 | 56,105.550 | | 21.6 | |  |
|  | Much | | 118 | 85,382.910 | | 32.8 | |  |
|  | Slightly | | 141 | 101,479.106 | | 39.0 | |  |
|  | Scarcely | | 27 | 16,961.513 | | 6.5 | |  |
| Economic Activity | Employment | | 170 | 131645.64 | | 50.4 | |  |
|  | Unemployment or economically inactive persons | | 187 | 129756.89 | | 49.6 | |  |
| Activity  Restriction | Yes | | 190 | 129614.7 | | 49.4 | |  |
|  | No | | 168 | 132834.2 | | 50.6 | |  |
