# Supplementary material for: Influence of Different Exercise Types on Health-Related Quality-of-Life in Men With Depressive Disorder in South Korea
Source: Front Public Health. 2022 Mar 14;10:811168. doi: 10.3389/fpubh.2022.811168 (PMC8964042; doi:10.3389/fpubh.2022.811168)
Supplement: Supplementary file 2 [file Table_2.docx]

**Table 2. The questions of research variables of each exercise type in KNHANES.**

| **Variable.** | | | **Question** | **Response Category** | |
| --- | --- | --- | --- | --- | --- |
|  |  |  |  | **Original** | **Modified** |
| **Independent Variable** | Flexibility Exercise | How many days did you perform flexibility exercises such as stretching etc. in the previous week? | | 1 - 6 | 1 - 4 |
|  | Strength  Exercise | How many days did you do strength exercises such as sit-up, push-up etc. in the previous week? | | 1 - 6 | 1 - 4 |
|  | Walking | How many days did you do walk at least 10 minutes at a time in the previous week? | | 1 - 8 | 1 - 4 |
