# Supplementary material for: Influence of Different Exercise Types on Health-Related Quality-of-Life in Men With Depressive Disorder in South Korea
Source: Front Public Health. 2022 Mar 14;10:811168. doi: 10.3389/fpubh.2022.811168 (PMC8964042; doi:10.3389/fpubh.2022.811168)
Supplement: Supplementary file 3 [file Table_3.docx]

**Table 3. The questions of research variables of each area in EQ-5D**

| **Variable.** | | **Question** | | **Response Category** |
| --- | --- | --- | --- | --- |
| **Dependent Variable** | Mobility | | I have in walking about. | 1 = no problem, 2 = some problem,  3 = am confined to bed |
|  | Self-Care | | I have with self-care. | 1 = no problem, 2 = some problem,  3 = am unable to wash or dress myself |
|  | Usual  Activity | | I have with performing my usual activities. | 1 = no problem, 2 = some problem,  3 = am unable to perform |
|  | Pain/Discomfort | | I have pain. | 1 = no pain, 2 = moderate,  3 = extreme pain |
|  | Anxiety/  Depression | | I am anxious or depressed. | 1 = not anxious, 2 = moderate anxious, 3 = extreme anxious |
