# Supplementary material for: Influence of Different Exercise Types on Health-Related Quality-of-Life in Men With Depressive Disorder in South Korea
Source: Front Public Health. 2022 Mar 14;10:811168. doi: 10.3389/fpubh.2022.811168 (PMC8964042; doi:10.3389/fpubh.2022.811168)
Supplement: Supplementary file 4 [file Table_4.docx]

| **Type of Exercise.** | **Participation**  **(Day)** | **Estimates** | | | | **SE** | ***t*** | | | | |
| --- | --- | --- | --- | --- | --- | --- | --- | --- | --- | --- | --- |
| Flexibility Exercise | 5 over | .048 | | | | .039 | 1.231 | | | | |
|  | 3-4 | .060 | | | | .045 | 1.317 | | | | |
|  | 1-2 | .032 | | | | .047 | .683 | | | | |
|  | Not once | .000 | | | |  |  | | | | |
| Strength Exercise | 5 over | .074 | | | | .026 | 2.806** | | | | |
|  | 3-4 | -.022 | | | | .038 | -.581 | | | | |
|  | 1-2 | .004 | | | | .031 | .116 | | | | |
|  | Not once | .000 | | | |  |  | | | | |
| Walking | 5 over | .024 | | | | .024 | 1.025 | | | | |
|  | 3-4 | .046 | | | | .031 | 1.480 | | | | |
|  | 1-2 | .102 | | | | .026 | 3.923*** | | | | |
|  | Not once | .000 | | | |  |  | | | | |
|  | | |  |  | R² = .401 *p* <.000 | | |  |  |  |  |

**Table 4. Outcomes of the influence of different exercise types (e.g., flexibility exercise, strength exercise, walking) on health-related QOL in men with DD in S. Korea.**

Note: ** *p* < 0.01, *** *p* < 0.001. Altered Variables: Activity restriction, age, degree of stress recognition, ownership of the house, economic activity.
